# Transcriptomes of Wet Skin Biopsies Predict Outcomes after Ionizing Radiation Exposure with Potential Dosimetric Applications in a Mouse Model

**DOI:** 10.3390/cimb44080254

**Published:** 2022-08-18

**Authors:** Abdulnaser Alkhalil, John Clifford, Stacyann M. Miller, Aarti Gautam, Marti Jett, Rasha Hammamieh, Lauren T. Moffatt, Jeffrey W. Shupp

**Affiliations:** 1 Firefighters’ Burn and Surgical Research Laboratory, MedStar Health Research Institute, Washington, DC 20010, USA; 2 Pain and Sensory Trauma Care Research Team, US Army Institute of Surgical Research, Fort Sam Houston, San Antonio, TX 78234, USA; 3 Medical Readiness Systems Biology Branch, Center for Military Psychiatry and Neuroscience Research, Walter Reed Army Institute of Research, Silver Spring, MD 20910, USA; 4 Department of Biochemistry and Molecular Biology, Georgetown University School of Medicine, Washington, DC 20010, USA; 5 Department of Surgery, Georgetown University School of Medicine, Washington, DC 20010, USA; 6 The Burn Center, Department of Surgery, MedStar Washington Hospital Center, Washington, DC 20010, USA

**Keywords:** radiation, transcriptomes, skin, mice

## Abstract

Countermeasures for radiation diagnosis, prognosis, and treatment are trailing behind the proliferation of nuclear energy and weaponry. Radiation injury mechanisms at the systems biology level are not fully understood. Here, mice skin biopsies at h2, d4, d7, d21, and d28 after exposure to 1, 3, 6, or 20 Gy whole-body ionizing radiation were evaluated for the potential application of transcriptional alterations in radiation diagnosis and prognosis. Exposure to 20 Gy was lethal by d7, while mice who received 1, 3, or 6 Gy survived the 28-day time course. A Sammon plot separated samples based on survival and time points (TPs) within lethal (20 Gy) and sublethal doses. The differences in the numbers, regulation mode, and fold change of significantly differentially transcribed genes (SDTGs, *p* < 0.05 and FC > 2) were identified between lethal and sublethal doses, and down and upregulation dominated transcriptomes during the first post-exposure week, respectively. The numbers of SDTGs and the percentages of upregulated ones revealed stationary downregulation post-lethal dose in contrast to responses to sublethal doses which were dynamic and largely upregulated. Longitudinal up/downregulated SDTGs ratios suggested delayed and extended responses with increasing IR doses in the sublethal range and lethal-like responses in late TPs. This was supported by the distributions of common and unique genes across TPs within each dose. Several genes with potential dosimetric marker applications were identified. Immune, fibrosis, detoxification, hematological, neurological, gastric, cell survival, migration, and proliferation radiation response pathways were identified, with the majority predicted to be activated after sublethal and inactivated after lethal exposures, particularly during the first post-exposure week.

## 1. Introduction

Radiation exposure in an accidental or intentional mass casualty event or occupational setup is a concern due to serious consequences to public health and military personnel [1]. Several molecules and cellular pathways of oncogenic nature are associated with radiation exposure [2,3,4]. Multiphasic acute radiation syndrome (ARS) frequently leads to organ failure and death [5] has been characterized [6]. Efforts to advance the understanding of host responses to radiation exposure rely on multiple animal models as well as case studies of workplace accidents or warfighter exposure. Numerous ex vivo human cell line radiation studies have explored cytogenic markers, nucleotide pool damage, and mutations [7] and showed significant changes in gene expression in response to radiation under heterogeneous experimental conditions [8,9]. Likewise, the limited radiation studies in animal models have been heterogeneous and demonstrated distinct changes in gene expression in various tissues [10,11]. 

Discovering countermeasures for radiation is challenging and has inherent limitations. To date, out of many tested drugs, only two, amifostine and granulocyte colony-stimulating factor (G-CSF), were approved for human use as a pharmacological radiation countermeasure by the US food and Drug Administration (FDA) [12,13]. The inability to obtain well-controlled human efficacy data as well as develop an animal model with satisfying translatability to humans are important hurdles facing the development of adequate radiation countermeasures [14]. Available biomarkers of radiation vary significantly in molecule type, efficacies, and ease of application. Examples include direct or indirect IR damages to DNA via reactive oxygen species (ROS) [15] and damage to bone marrow in hematopoietic subsyndrome (H-ARS). Similarly, many small non-protein molecules, such as citrulline, and proteins functioning as cytokines and chemokines, including granulocyte colony-stimulating factor (G-CSF) [16,17,18,19,20], interleukin-6 and 18 (IL6, IL18) [21], serum amyloid A (SAA), C-reactive protein (CRP) [22], FMS-like 3 ligand (flr3L) [23], and growth arrest and DNA damage-inducible 45 (GADD45) [13,24], were suggested as radiation biomarkers. Measurable changes in counts of peripheral blood cells after radiation exposure were proposed as biomarkers [24,25] with limited applicability due to measurement variation over a wide time window (2 days–4 weeks) and the large dose required to elicit the response (>6 Gy) [25]. The investigation of chromosomal aberrations that were observed after radiation [26] introduced di-centric chromosomes in association with tailed nuclei as a test for radiation exposure [27,28]; however, this method is unsuitable for mass casualty events due to the skilled examiners required and its laborious nature. Other radiation exposure studies have explored cytogenic markers, nucleotide pool damage, and mutations [29]. Extensive work has examined the phosphorylation of histone H2AX at the site of DNA double-strand breaks and the consecutive accumulation of H2AX-γ as a biomarker of such DNA damage. The relationship between H2AX-γ protein modification and low-dose radiation exposure was validated with potential for biomarker applications [30,31,32]. However, most of the published methods for H2AX modifications involve labor-intensive and non-rapid methods employing immunocytochemistry and immunohistochemistry (IHC) approaches [33,34]. Additional work in ex vivo human cell lines under heterogeneous exposure conditions (radiation source, dose, and time of analysis) [8,9,35] led to unclear dose- and time-dependent responses. Moreover, miRNA analyses showed only three miRNAs were altered after high linear energy transfer (LET) irradiations, while six miRNAs were altered after low LET irradiations [36,37]. Fewer published studies have used epigenetics to examine DNA modification, specifically methylation, which is a likely target of environmental exposures. Studies reported increased or decreased methylation in response to ionizing radiation [36,37,38], but results varied and did not identify a specific useful biomarker [39]. Despite the many unsuccessful or unvetted proposed biomarkers, advances in the analysis of microbiomes, metabolomes, and transcriptomes have been recognized as promising approaches for identifying reliable radiation markers [40,41,42]. Particularly, transcriptomic profiling has become a powerful tool for discovering biomarkers for quicker diagnoses and a better understanding of post-exposure survival and death mechanisms that support the discovery of novel therapies or intervention strategies [43]. To gain detailed insight into the immediate effects of ionizing radiation (IR) on the skin and search for biomarkers with biodosimetry applications, we conducted a radiation dose–response and time-course experiment in mice that included an assessment of the complement of transcribed genes (transcriptome) of the skin. 

## 2. Materials and Methods

### 2.1. Ethics

Mice were handled according to the facility’s standard operating procedures under the animal care and use program accredited by the Association for Assessment and Accreditation of Laboratory Animal Care International (AAALAC) and Animal Welfare Assurance through the Public Health Service (PHS). All described animal work was reviewed and approved by our Institutional Animal Care and Use Committee (IACUC). The research described in this work adheres to principles stated in the Guide for Care and Use of Laboratory Animals, NRC Publication, 2011 edition.

### 2.2. Animal Radiation Model and Sample Collection 

Eight-week-old male C57BL/6 mice purchased from The Jackson Laboratory (JAX, Bar Harbor, ME 04609 USA) were acclimated for one week before initiating IR exposures and specimen collection. All animal work was performed and completed at animal age < 14 weeks. Animals were housed at a density of five mice per cage under standard housing conditions of food, temperature, water, and 12/12 h light/dark cycles. 

### 2.3. Radiation Treatment

Mice were placed individually in a pie-shaped round container divided into five equal size triangular compartments pointing to the center of the container covered with the same vented lid. The container was placed under a linear accelerator (Clinac 2100EX Manufacturer: Varian Medical) with a field size set at 32 × 32 cm to ensure the coverage of the whole container. Based on the dose needed, monitor units (MUs) were calculated and delivered half from the anterior and half from the posterior (standard AP/PA technique). The energy of the beam used to deliver the dose was 6 MV photon and ran at a dose rate of 600 MU per minute. The machine output was calibrated following the TG51 protocol [44]. Mice received whole-body X-ray exposures (0, 1, 3, 6, or 20 Gy) while under anesthesia using the IP injection of 300 μL of ketamine (3 mg) and 50 μL of xylazine (3 mg) in saline. Isoflurane (2–5%) was used in a controlled gas flow box or through a nose cone for anesthesia maintenance as needed. The whole-body X-ray doses were selected to mimic scenarios in which a population is exposed to radiation doses spanning the range of unsalvageable lethal to survivable exposures with minimal medical intervention in humans to collect information concerning response enhancement and ultimately improve the survival rate in a mass casualty event.

### 2.4. Samples Collection and Post-Irradiation Observation

Animals returned to the housing facility and were kept until skin biopsies were collected from each animal at hour 2 (d0), d4, d7, d21, and d28 post-irradiation. Briefly, the animals’ dorsa were shaved using standard veterinary clippers, and a 1 cm2 biopsy from each animal was collected while the animal was under anesthesia, as described above. Biopsy sites were closed using prolene sutures (Ethicon, Johnson & Johnson, New Brunswick, NJ, USA). Animal observation until the completion of the full experiment time course showed no signs of pain or distress after exposure to 0, 1, 3, or 6 Gy or biopsy. Mice exposed to 20 Gy showed decreased activities by post-exposure day 6 and developed signs of distress, lethargy, and dehydration by day 7. Mice in the latter IR exposure dose were euthanized on the same day when signs of distress were confirmed per humane endpoint criteria defined in the IACUC-approved study protocol. Animals in the mock-radiation exposure (sham) condition were handled and housed identically to the IR-exposed animals, in which the mice were loaded in the same container and transported to the radiation facility and returned under the same conditions with no radiation applied, anesthetized, and shaved for biopsy collection. At the end of the experiment time course, euthanasia was performed via exsanguination using cardiac puncture under anesthesia. Death was confirmed by the lack of pedal and corneal reflexes and the opening of the thoracic cavity to ensure the lack of heartbeat. Some of the mice groups in the 20 Gy radiation experiment arm were moribund before the targeted endpoint. Animals were terminated when signs of acute radiation syndrome, including diarrhea and weight loss, were observed regardless of the intended endpoints.

### 2.5. Molecular Biology

RNA extraction: Total RNA was isolated from liquid nitrogen flash-frozen biopsies from each animal after being ground in a cold mortar and pestle. A Porcelain Mortar, size 0, 50 mL, 70 mm diameter, Coors, 60310 and a Porcelain Pestle, size 0, 114 mm long, Coors, 60311 (Porcelain Mortars and Pestles. CoorsTek, Manufacturer: Coorstek, Family Part #: COORS TSI-603) were used to grind the tissue for RNA extraction. Each grounded biopsy was transferred into a 1.5 mL tube containing 1 mL of TRIzol reagent (Invitrogen, Thermo Fisher, Waltham, MA, USA), and RNA was isolated following the manufacturer’s protocol. The concentrations and quality of yielded mRNA were assessed using NanoDrop 1000 (Thermo Fisher, Waltham, MA, USA) and the Agilent 2200 Tapestation system (Agilent Technologies, Santa Clara, CA, USA). The material was aliquoted and stored at −80 °C until further use. 

cDNA Microarray and Processing: 25–200 nanograms of RNA was used following Agilent’s two-color array workflow utilizing Low Input Quick Amp Labeling Kit, Two-Color, RNA Spike-In Kit, Two-Color, Gene Expression Hybridization Kit, and Gene Expression Wash Buffer Kit (Agilent Technologies, Santa Clara, CA, USA), following the manufacturer’s instructions. Briefly, samples and purchased reference RNA (Agilent Technologies) were reverse transcribed and labeled with Cy-5 and Cy-3 dyes, respectively. All samples were then purified using the RNeasy Mini Kit (Qiagen) and quantified on Nanodrop. Both labeled cDNAs were simultaneously hybridized for 17 h at 65 °C on the Agilent 4 × 44K Whole Mouse Genome Microarray Kit (GPL7202: Agilent-014868); then, slides were washed (Agilent Technologies, Inc., Santa Clara, CA, USA). Arrays were immediately scanned using an Agilent G2505C Scanner (Agilent Technologies Inc., Santa Clara, CA, USA).

### 2.6. Data Preparation and Analysis

Images were processed using Agilent’s default Feature Extraction software v11.0.1.1 and analyzed using custom R scripts to obtain lists of probe sets differentially expressed. Minimum information about a microarray experiment (MIAME)-compliant intensity, quality, and normalized ratio data for this series of experiments were deposited in the gene expression omnibus (GEO) database maintained by the National Center for Biotechnology Information (accession no. GSE185149). Changes in gene expression at Benjamini–Hochberg FDR-adjusted *p* < 0.05 were considered in identifying the significance of transcription modulation. Analyses were performed comparing the different doses of X-ray exposure relative to the control unexposed mice over all TPs. Uncentered Pearson clustering was carried out with tools developed by the Division of Computational Bioscience of the Center for Information Technology and the Cancer Genetics Branch of the National Human Genome Research Institute at the NIH. 

Samples that failed in RNA extraction or had a low RNA integrity number (RIN < 6) were removed from the microarray experiment. All microarray data were preprocessed, and quality control was performed using within-chip Lowess and between-chip quantile normalization. Outliers were identified using Principal Component Analysis and were removed from the downstream analysis. Significantly differentially transcribed genes (SDTGs) were identified using the Bioconductor limma 3.7 package [45]. The fold change (FC) was defined by the average log2 expression of the radiation group minus the average log2 expression of the control group. Further refinement of the SDTG lists was performed by sorting the gene lists passing *p*-value < 0.05 based on the FC and selecting top genes meeting the absolute value of FC > abs 2 and *p*-value < 0.05. 

Ingenuity Pathway Analysis (IPA): Lists were crossed with lists of annotated SDTG lists obtained after loading to Ingenuity Pathway Analysis (QIAGEN Inc., https://digitalinsights.qiagen.com/IPA (accessed between 1–15 December 2021)). Only genes that were common to both lists were included in all subsequent analyses. The top pathways are reported based on abs z scores from IPA. Lists of genes in the reported pathways were obtained from IPA with no further processing of additional cutoffs. Analyses were performed comparing the results of significantly differentially regulated genes after exposure to different doses of X-ray over several TPs. The analysis of networks and pathways for differentially transcribed genes (*p* < 0.05 and FC ≥ 2) between 20 Gy and 0 Gy at day 0, 2 h, d4, d7, d21, and d28 identified the biological functions and/or diseases that were most significantly relevant. Those gene networks and their associated biological functions and/or diseases were summarized and presented as lists or heat maps after applying z-score filters (≥Abs 2). The bystander response due to serial biopsying among TPs was minimal in the sham group and was subtracted in the comparative analysis of responses in the irradiated groups. 

## 3. Results

**Figure 1:**
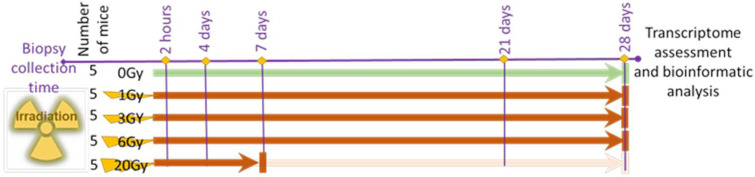
Study design. Five groups each consisting of five animals received 0, 1, 3, 6, or 20 Gy of whole-body X-ray irradiation. Animal skin biopsies were collected at 2 h and day 4, 7, 21, and on euthanasia day 28. Animals exposed to 20 Gy did not complete the study time course and were euthanized on day 7.

Seeking a detailed insight into the effects of IR on the skin and to identify transcription trends or gene candidates with potential biomarker applications in a dosimetry tool, five groups of five young mice each were exposed to an X-ray radiation dose of 0, 1, 3, 6, or 20 Gy, and skin biopsies from each animal were collected at 2 h, 4, 7, 21, and 28 days after exposure (Figure 1). All mice exposed to 20 Gy showed distress signs by day 7 and were euthanized under the humane endpoint criteria in the IACUC-approved protocol of the study (Figure 1). All mice exposed to lower IR doses survived the 28-day time course of the experiment. Thus, the terms lethal and sublethal are used in this report to refer to the 20 Gy and the rest of the IR doses, respectively. The transcriptome profiles of skin biopsies from mice in all IR exposures were interrogated using microarrays, and data were analyzed. 

### 3.1. Skin Transcriptomes Predict the Short-Term Fate of Mice after IR Exposure

**Figure 2:**
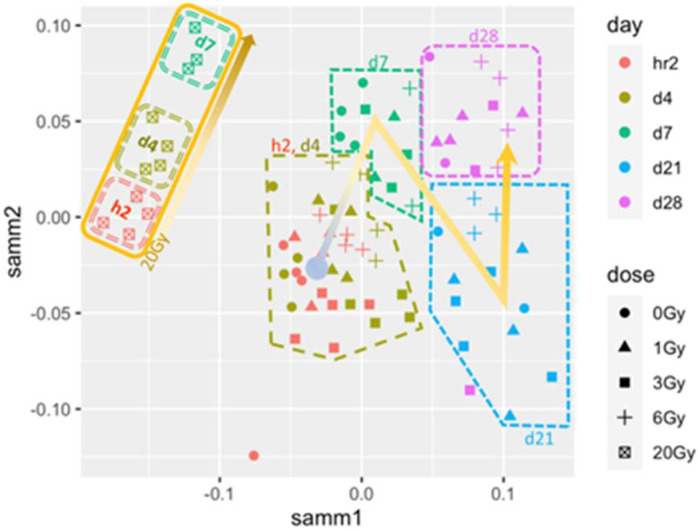
Sammon plot analysis showing separation of samples based on irradiation levels and time after exposure using transcriptomics.

The initial analysis of all transcriptome data indicated sample clustering along both time and dose when viewed in a Sammon mapping plot (Figure 2). The largest separation was noticed between samples from mice that had received a lethal dose (20 Gy) and those of the sublethal doses (i.e., 1, 3, and 6 Gy). Biopsies from animals exposed to the lethal dose (BLD) formed a distinctive aggregate containing three clusters each consisted of samples of the same TP. These sample clusters synchronously transitioned linearly from 2 h to d4 to d7 across the two dimensions of the Sammon plot (Figure 2). Biopsies from animals exposed to sublethal radiation doses (BSDs) (i.e., 1, 3, or 6 Gy) clustered more according to biopsy day (i.e., time points) than exposure dose, suggesting a level of similarity in the response to sublethal radiation doses that progresses among TPs.

**Figure 3:**
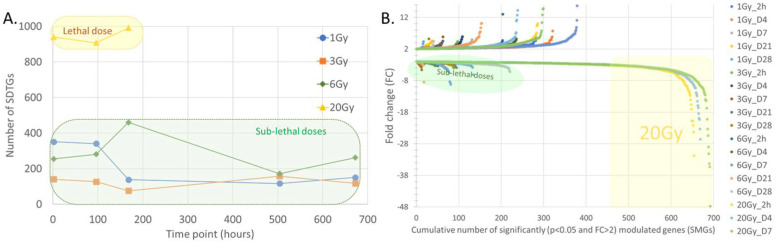
Longitudinal profile of significantly differentially transcribed genes (SDTGs, *p* < 0.05 and FC ≥ 2) after irradiation with 1, 3, 6, or 20 Gy. Dynamics of SDTGs during the experiment time course (**A**). Dot plot showing the number and magnitude of the regulation (up- and downregulated genes are separated) in SDTGs at all doses and time points (**B**).

Several thresholds were tested to optimize the selection of the significantly differentially transcribed genes (SDTGs). A stringent cutoff of a *p*-value < 0.05 and fold change (FC) ≥ 2 were adopted to accept elements in all subsequent analyses. The number of genes meeting these criteria varied significantly among exposure levels (Figure 3), supporting radiation-dose-dependent responses. In agreement with observations from the Sammon plot, transcriptional modulations after lethal exposure were much larger in numbers of genes (Figure 3A, B) and the magnitude of regulation relative to sublethal exposures (Figure 3B) in all TPs, suggesting different immediate responses to lethal and sublethal exposures. 

### 3.2. Idling Transcription Is a Principal Response Character in BLDs Contrary to That in BSDs 

**Figure 4:**
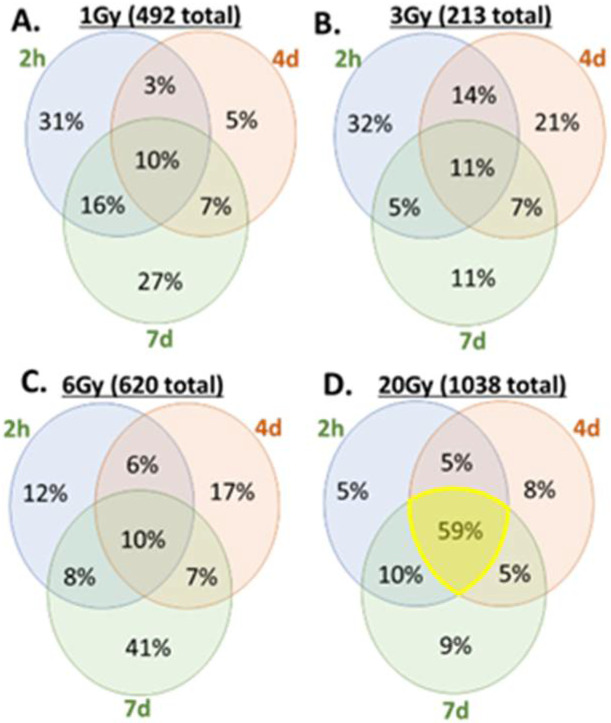
Venn diagrams for SDTGs’ distribution among the time points of BLDs (**D**) and corresponding BSD (**A**–**C**) time points (i.e., h2, d4, and d7).

Evidence for differences in transcriptional responses was further supported by the comparison of the distribution of SDTGs among the first three TPs (2 h, 4d, and 7d) in BLDs and BSDs. About 59% of the total SDTGs identified in BLDs were common to all three TPs (Figure 4D), while less than 11% were common among the same TPs in BSDs (Figure 4A–C). The contrast between the stationary transcriptional regulation in BLDs (20 Gy) and the active one in BSDs (1, 3, and 6 Gy) presents a valuable method to distinguish between lethal and sublethal IR exposures in vivo. The large difference in the transcriptional regulation state between BSDs and BLDs suggests a dose-dependent switch in the 6–20 Gy range where transcription transitions to a stationary state.

### 3.3. Transcription Regulation Dynamics and Mode of Gene Regulation Differentiate among Sublethal IR Doses 

**Figure 5:**
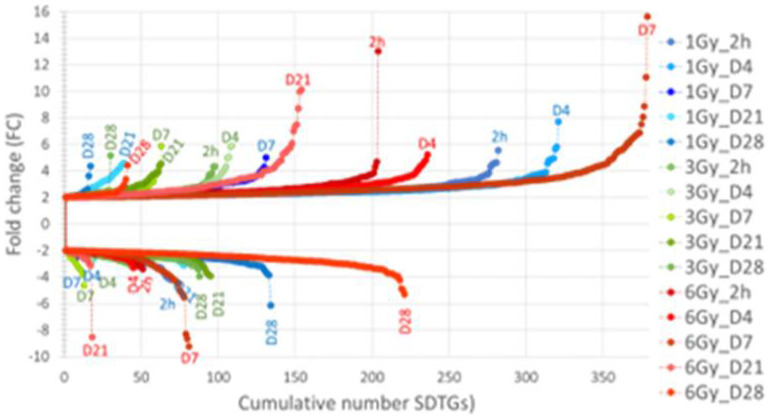
Dot plot showing the number and magnitude of up- and downregulation of SDTGs (*p* < 0.05 and FC ≥ 2) in skin biopsies of mice exposed to different to sublethal doses of X-ray radiation. Note the varied patterns between up- and downregulation in each of the three doses and among all three doses.

**Figure 6:**
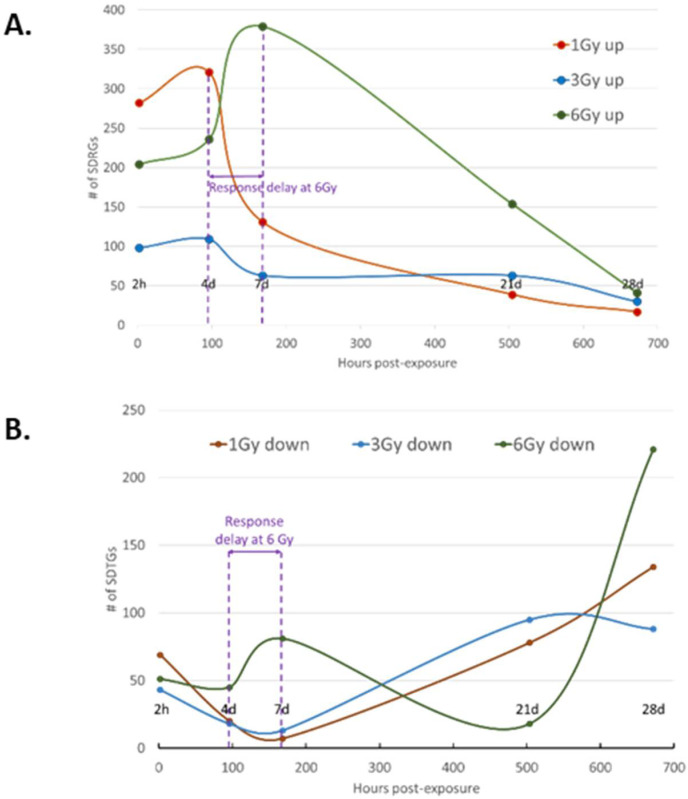
Regulation pattern variation in SDTGs in skin biopsies of mice exposed to sublethal doses (1, 3, and 6 Gy) of X-ray radiation during the study time course. Patterns in upregulated SDTGs (**A**) and downregulated SDTGs (**B**). Note the peak in upregulation and troughs in downregulation coincided at 1 and 3 Gy doses, while downregulation at 6 Gy seemed to have more than one phase.

**Figure 7:**
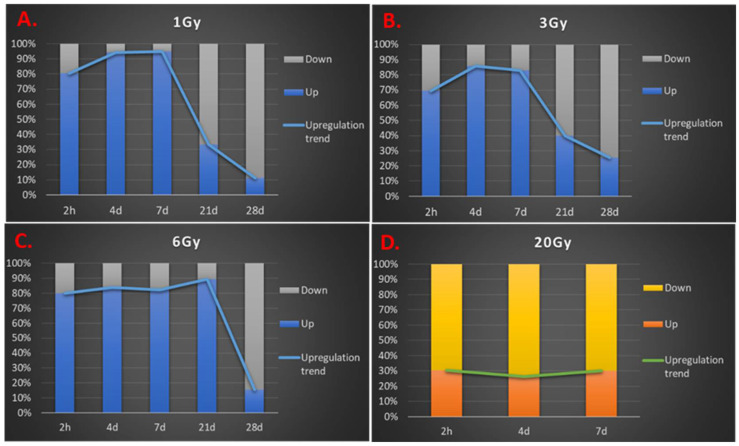
Transcriptional regulation in skin biopsies from mice exposed to different IR doses expressed as ratios of up- and downregulation percentages. Note the inverse relationship of transcription regulation in BLDs and BSDs and shifts of response peaks to later time points by increasing doses of radiation among BSDs.

In addition to the difference in the numbers and FC values of SDTGs between BLDs and BSDs, transcription modulation was skewed largely to downregulation in BLDs (Figure 3B) and upregulation in BSDs (Figure 5), especially during the first three TPs, where the number of SDTGs and the intensity of regulation peaked (Figure 5). The number of upregulated and downregulated genes peaked at D7 and D28 for 6 Gy exposures, D4 and D21 for 3 Gy exposures, and D4 and D28 for 1 Gy exposures (Figure 5). The most upregulation and downregulation FCs were observed at the highest IR exposure in the BSD (6 Gy). However, the radiation-dose-dependent trend in the number and magnitude of SDTGs seen at 20 and 6 Gy (Figure 3 and Figure 5) did not apply at lower radiation doses, and larger numbers of SDTGs were found up- and downregulated at 1 Gy relative to 3 Gy in many TPs, which suggested the involvement of additional factors in transcription regulation at these doses. Interestingly, no single pattern for up- and downregulation could be recognized at all IR doses (Figure 6), and while upregulation peaked at post-exposure day 4 in the 1 and 3 Gy doses, the peak was delayed until day 7 in the biopsies of animals exposed to 6 Gy (Figure 6A). Moreover, the numbers of downregulated SDTGs in 1 and 3 Gy decreased early during the first three TPs while remaining steady and then increasing at d7 in the 6 Gy during the same duration (Figure 6B). The difference in up- and downregulation responses may potentially differentiate among radiation doses within BSDs, where responses gradually acquire more features of a BLD as IR dose increases. These features include an increased number of downregulated genes in the early TPs and a shift to a more delayed and stagnant response. It is important to note that responses at later TPs in all BSDs show a slow increase in the numbers of downregulated SDTGs, which seems to be steeper at the higher IR dose of 6 Gy (Figure 6B). The dynamics of the up- and downregulated SDTGs’ relationship were elucidated when expressed as the percentage of upregulated SDTGs in the total number of SDTGs at each TP (Figure 7). The inversed direction of regulation in BLDs and BSDs (Figure 7D vs. A–C) and the delay in response with increasing IR doses within BSDs (peak shifts in Figure 7A–C) are also demonstrated. 

### 3.4. Distribution of SDTGs through the Time Points and in Different IR Doses Distinguishes Responses in Lethal and Sublethal IR Doses

Tracing SDTGs (*p* < 0.05 and FC > 2) by their identities at different TPs of the same IR dose showed that the percentage of unique SDTGs to any TP in BLDs did not exceed 9%, and most of the genes were common to all TPs (59%), underscoring the stationary nature of the response at high IR doses (Figure 4D) relative to that in BSDs, where the largest fractions of SDTGs were TP-specific (Figure 4A–C). An inversed relationship was observed between the increased IR dose and the unique/common SDTGs ratio during the first TP after exposure (3.1875, 2.833, and 1.2833 in 1, 3, and 6 Gy, respectively, versus 0.095 in 20 Gy at h2). The largest response ratio in the 6 Gy dose was at d7 (unique/common = 4.1), while it coincided earlier (h2) in 1 Gy and 3 Gy, confirming the delay in responses with the increasing IR doses, as observed in the general regulation analysis above (Figure 3 and Figure 6). 

The global analysis of SDTGs’ (*p* < 0.05 and FC > 2) identities showed that a total of 1665 genes were modulated throughout the whole study (Table 1). Each SDTG was modulated in at least one TP in one of the four IR doses (1, 3, 6, or 20 Gy). No gene was found to be common among all doses and TPs (Table 1). The lack of common genes indicated highly diverse time- and dose-dependent responses. The distribution of TP-unique genes reproduced the same peak response time seen in the quantitative regulation analysis in BSDs, and the relatively small numbers of TP-unique SDTGs in BLDs confirmed the static character of regulation at the 20 Gy dose again (Table 1). The idle gene transcription in BLDs was further exposed when the analysis was performed to compare TPs in each IR dose independently (Table 2), where the numbers of common genes to all TPs within each dose were by far largest in BLDs (20 Gy) than that in BSDs (1, 3, 6 Gy), and the number of TP-unique SDTGs was smaller than that in many TPs in BSDs despite the lower number of total SDTGs at BSD doses (Table 2). The peaks of the number of the TP-unique SDTGs supported delayed responses in the larger IR dose amongst the sublethal doses (Table 2). The Definsin β6 (Defb6) gene, which encodes a protein that binds a CCR6 chemokine receptor and exhibits chemoattractant activity, was the only gene common to all TPs of the 1 Gy dose and was downregulated at 2 h and d4 then upregulated at d7, d21, and D28. The modulation of Defb6 transcription was not exclusive to the 1 Gy dose and was also found significantly modulated at a couple of TPs in larger IR doses. Four genes were common to all TPs after the 6 Gy exposure. Those genes were the abhydrolase domain containing 3 phospholipase (ABHD3), which was upregulated in all TPs, the ChaC glutathione specific gamma-glutamylcyclotransferase 1 (CHAC1), which was downregulated in all TPs, the carbonic anhydrase 3 (CA3), and the major urinary protein 1 (Mup1). The latter two genes were downregulated at d28 and upregulated in all other TPs. No gene was found to be common in TPs after the 3 Gy exposure, and a large number of mainly downregulated genes were common to the three TPs after the 20 Gy dose. A list of the top SDTGs (*p* < 0.05 and FC > 3.5) that were unique to each TP within an indicated IR dose is included in Table 3. In agreement with the predominant downregulation of the SDTGs in the BLD, the three genes for the 20 Gy in Table 3 were downregulated. Similarly, most of the SDTGs in the 1 Gy in the table were upregulated, and the numbers of SDTGs supported the shift in the peaks of response to later TPs with the increasing dose of IR among BSDs and the gradual increase towards more downregulation in the last TP of the study.

### 3.5. Response Dynamics Are More Time-Based Than Dependent on IR Dose in the Sublethal Dose Range 

The distribution of SDTGs at the same TP for all IR doses showed that 17, 24, 16, 5, and 19 genes were common to all IR doses at h2, d4, d7, d21, and d28, respectively (Table 4). The top TP-common SDTGs (*p* < 0.05, FC > 2 and average Abs FC > 3 in all TPs) across all doses are listed in Table 5. Almost all the genes in the table were upregulated at all TPs in BSDs and downregulated in the BLD, once more confirming the overwhelming staggered character of regulation trends in BSDs and BLDs. The intensity of regulation increased by the increase in received IR dose in most of the SDTGs. Two genes encoding the actin-binding Rho activating protein (ABRA) and the S100 calcium-binding protein A9 (S100A9) were upregulated in BSDs and BLDs in all IR doses at 2 h and d4, respectively. Only two TP-common SDTGs (*p* < 0.05, FC > 2 and average FC > 3 in all TPs) were found downregulated at all BSDs at d28. The larger numbers of TP-common SDTGs (Table 4) relative to the numbers of SDTGs common to all doses at a specific TP (Table 2) suggests a differential transcription that progresses with time faster than being defined by IR dose intensity in the sublethal dose range (Table S1). This trend was reversed in the lethal dose, where common SDTGs were largest among different TPs of the lethal dose than with the same TP in other IR doses. 

Larger numbers of SDTGs with the highest FCs, the longer modulation dwell-time spanning multiple consecutive TPs, and the predominant downregulation of the SDTGs are all indicative of exposure to lethal IR doses. The more time-point-specific SDTGs, the earlier the peak of the response, and the higher the ratio of upregulation/downregulation, the lower the IR dose exposure. 

### 3.6. Specific Significantly Differentially Transcribed Genes Distinguish between Exposure to Lethal and Sublethal IR Doses

A total of 1499 genes were significantly differentially transcribed (*p* < 0.05 and FC > 2) in lethal and sublethal IR doses during the h2, d4, and d7 TPs. The largest number of IR-dose-unique SDTGs was associated with the lethal dose exposure (Table 6) in each TP, confirming the general trend observed in global analysis. 

The numbers of IR-dose-unique SDTGs in the sublethal doses showed the highest number was at h2 after the 1 Gy, while it was at d4 and d7 after 6 Gy exposure, again, underscoring the same trends of earlier peaks of radiation response at lower IR doses within the sublethal dose (Table 6). To identify genes that can potentially be applied in detecting radiation exposure and distinguishing lethal from sublethal dose exposures, lists of the SDTGs at the first post-exposure week (i.e., h2, d4, and d7) in lethal and sublethal doses were compared. Out of 1499 transcriptionally modulated SDTGs (*p* < 0.05 and FC > 2) in all doses and TPs, 609 were common in all lethal (20 Gy) TPs, 390 of which were unique to the lethal dose only and were not found in any sublethal IR doses during the same duration. Only ten genes were identified as common to all three TPs of the sublethal exposures (1, 3, and 6 Gy), in support of vigorous and dynamic responses in the sublethal IR doses. The difference in the number of common genes to lethal (390 genes) and sublethal (10 genes) reiterates the opposing IR dose-based transcriptional responses in lethal and sublethal exposures. Out of the common 10 genes at all three TPs of the sublethal IR doses, only 3, namely cystatin A (CSTA), cytochrome P450, family 2, subfamily b, polypeptide 9 (Cyp2b13/Cyp2b9), and hornerin (Hrnr), were unique to sublethal doses and not involved in the response to the lethal 20 Gy dose. All three genes were found upregulated at h2, d4, and d7 in the sublethal IR doses. Equally important was the identification of five genes that were commonly significantly differentially transcribed in the lethal and sublethal IR doses during the h2, d4, and d7 TPs (Table 7) and were consistently downregulated in the lethal dose and upregulated in all sublethal IR doses at all TPs. 

### 3.7. Pathway Analysis Revealed Inverted Biological Responses in the BLDs and BSDs at All TPs

**Figure 8:**
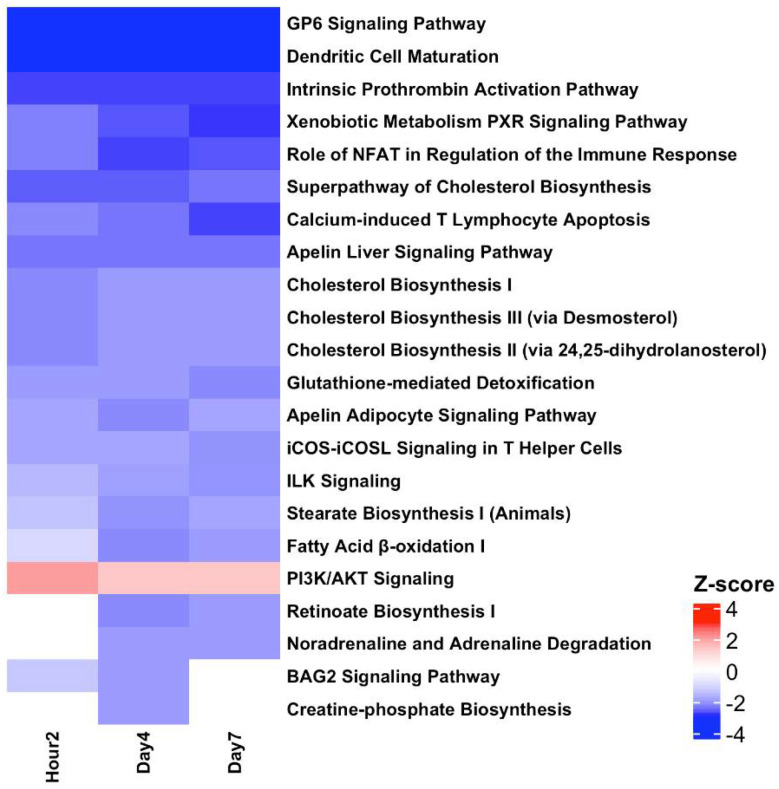
Significantly (Abs z-score ≥ 2 and −log *p* ≥ 1.3) modulated pathways in at least one time point from the analysis of SDTGs (*p* ≤ 0.05 and Abs FC ≥ 2) after lethal IR dose exposure at all TPs.

Pathway enrichment of the SDTGs (*p*-value < 0.05 and FC > 2) at each TP in each IR dose was performed to identify significantly affected pathways and biological functions. The first phase of analysis focused on comparing modulated pathways in biopsies from mice exposed to the lethal IR dose. A total of 22 pathways were significantly (Abs z-score ≥ 2 and −log *p* ≥ 1.3) modulated at h2, d4, and d7 TPs after exposure to 20 Gy, of which 12 pathways were modulated at all three TPs (Figure 8). All modulated pathways were inactivated, except for the PI3K/AKT signaling pathway, which was predicted to be activated at the h2 TP alone (Figure 8). The comparison of these pathways with data from the same three TPs in sublethal dose exposure showed that out of the 22 pathways that were found significantly modulated at the lethal 20 Gy IR dose, none shared a common prediction of the activation status, and 11 of these pathways were unique to the 20-Gy-exposed samples, while the other 11 pathways showed the opposite activation status in sublethal doses (i.e., active) in at least one TP. Most of the pathways of sublethal doses were modulated at d4 for 1 Gy and d7 for 6 Gy, in agreement with the response shift to a later TP with increasing IR doses. None of the pathways that were predicted to be significantly modulated after lethal dose exposure showed an inversion of the activity status throughout all survived TPs (h2, d4, and d7). The comparison of modulated pathways in the 1, 3, and 6 Gy exposure versus the 20 Gy exposure showed that a total of 62 pathways were significantly (Abs z-score ≥ 2 and −log *p* ≥ 1.3) modulated in at least one TP (h2, d4, or d7) in one of the IR doses (1, 3, 6, or 20 Gy). The 62 pathways showed 158 counts of a pathway being significantly modulated (Abs z-score ≥ 2 and −log *p* > 1.3) in a specific TP. Almost half of the incidents (80/158 or %50.6) were for inactivation predictions (z-score ≤ −2), and 90% of them (72/80) were associated with 20 Gy exposure (Figure S1), while the other eight inactivation incidents (10%) were associated with the three sublethal IR doses (Figure S1). The 78 pathway-activation incidents showed an almost opposite trend where 77/78 (%98.71of the total) were associated with TPs after sublethal exposure, and only one incident predicted an activation. 

**Figure 9:**
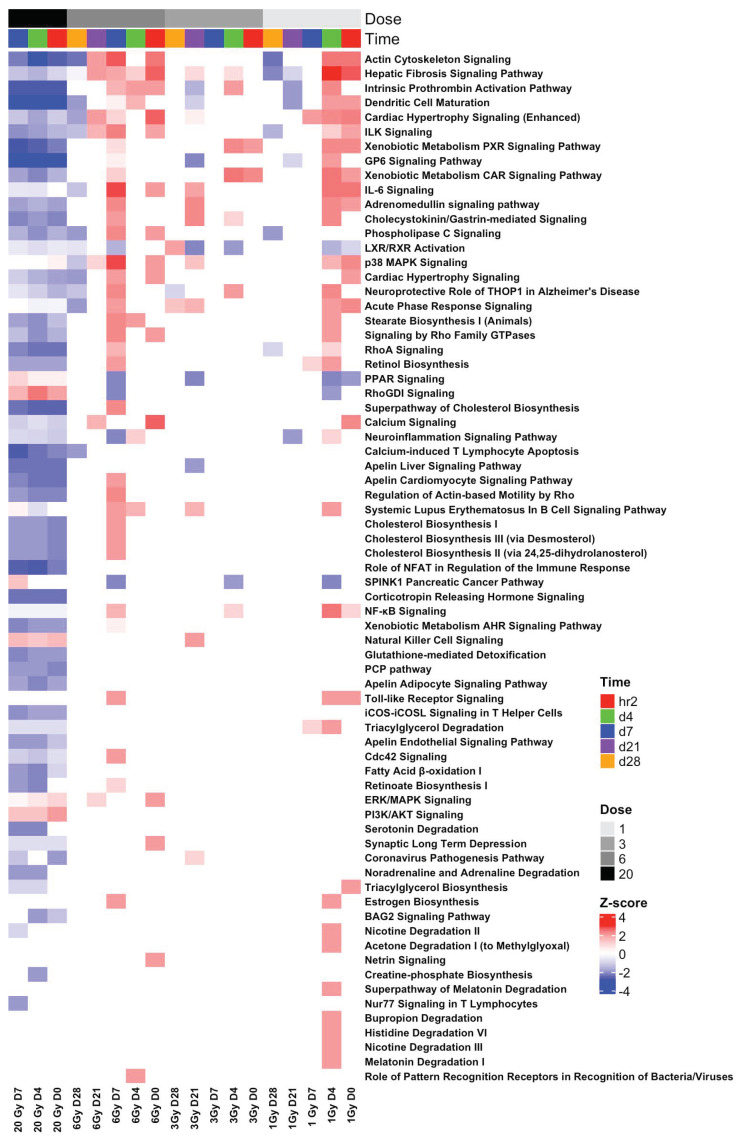
Significantly (Abs z-score ≥ 2 and −log *p* ≥ 1.3) modulated pathways in at least one time point from the analysis of SDTGs (*p* ≤ 0.05 and Abs FC ≥ 2) in all TPs and all IR doses. A total of 71 pathways were identified to be modulated at 202 assessment points, of which, 107 were inactivation and 81/107(or 76%) were associated with TPs of the 20 Gy exposure. Blue color for inactivation and orange for activation prediction. Color darkens with increased modulation intensity.

The addition of the d21 and d28 TPs of the sublethal (1, 3, and 6 Gy) doses to the comparison to assess the extended response in sublethal doses, using pathway activity predictions in the lethal dose as a reference, increased the number of the significantly (z-scores > abs 2, and −log *p* > 1.3) gene-enriched pathways from 62 to 71 and increased the counts of a pathway being significantly modulated (Abs z-score ≥ 2 and −log *p* > 1.3) in a specific TP from 158 to 202, 107 (%52.97) of which were inactivation and 81/107 (%75.7) were associated with TPs of the lethal dose. The number of pathways that were predicted significantly (z-scores > abs 2 and −log *p* < 1.3) activated increased from 78 to 96, and 94 of which (94/96 or%97.91) were associated with sublethal doses. Only two incidents of the activated RhoGDI signaling pathway at d4 and the PI3K/AKT pathway at h2 were significant after lethal dose exposure. Eight of the nine additional pathways were either predicted to continue the same opposing activity trend seen in earlier TPs relative to activity in lethal doses (apelin cardiomyocyte signaling, RhoGDI signaling, and signaling by Rho family GTPases), or were unique to lethal (the PCP pathway and corona pathogenesis pathway) or sublethal doses (cardiac hypertrophy signaling, natural killer cell signaling, and systemic lupus erythematosus in the B-cell signaling pathway). Only one pathway, the phospholipase C signaling pathway, showed common regulation in the lethal dose at d4 and sublethal doses 1 and 6 Gy at d28 (Figure 9). Six other pathways (intrinsic prothrombin activation, GP6 signaling, calcium-induced T lymphocyte apoptosis, dendritic cell maturation, actin cytoskeleton signaling, and apelin liver signaling), which were already identified among the significant sixty-two pathways in the earlier three TPs of sublethal doses and showed opposing regulation with the lethal dose–response, showed inversion in activity prediction at D21 or D28 and hence simulated the predicted activation status in the lethal 20 Gy dose (Figure 9). The inversion of the activation mode of these pathways from opposing to similar relative to the lethal in the late sublethal TPs is indicative of a potential delayed damaging IR effect. Out of the 71 pathways, 27 were found significant in either lethal or sublethal doses and negligible in the other. All other pathways were either unique to lethal (14 pathways) or sublethal doses (10 pathways) or were predicted to have a significant and opposing activation status in lethal and sublethal doses (20 pathways predicted inactive in lethal and active in sublethal or vice versa). The number of unique pathways increased from 14 to 18 and 10 to 33 in lethal and sublethal doses, respectively, when non-significant z-scores for the pathways were ignored. Unlike pathway modulation after lethal dose exposure, pathways identified at sublethal doses rarely dwelled for more than two consecutive TPs (Figure 9). 

All significantly identified pathways were modulated oppositely in lethal and sublethal doses, with the vast majority being inactivated at a lethal dose (20 Gy) and either activated or not present at the sublethal doses (1, 3, and 6 Gys), and only the RhoGDI signaling and the PIK/AKT signaling pathways were found to be activated at d4 and 2 h, respectively, after the lethal dose and were predicted inactivated or not present at sublethal TPs.

## 4. Discussion

The increasing use of radioactive materials in therapies, energy generation, and the proliferation of nuclear devices elevates the risks of public radiation exposure. Identifying radiation exposure and estimating the absorbed dose is essential in guiding effective treatment and efficient triage in the cases of mass casualty events. Providing appropriate care is complicated by the variation in absorbed IR dose due to shielding effects and the asymptomatic early phase of exposure even when receiving a life-threatening dose. 

Despite the recent progress in developing IR exposure diagnostics, biomarkers, and therapeutics, the management of IR exposure remains a healthcare challenge, and adequate radiation countermeasures and a clear understanding of radiation pathogenesis are still lacking. This report is a contribution to the ongoing endeavors to address these countermeasure shortages. 

In agreement with previous studies, exposure to 20 Gy in this work was lethal to mice within a week. Mice exposed to lower doses of 1, 3, or 6 Gy completed the experiment time course (28 days), and no noticeable adverse symptoms were observed in these sublethal mice exposures. The analysis of transcriptomics in BLDs and BSDs using a Sammon plot that included all elements in the microarrays distinguished samples from animals exposed to lethal and sublethal IR doses by separating BLDs and BSDs into two distinct clusters indicating dissimilar responses. Samples within the BLDs were also separated, but to a lesser degree than separation from BSDs, based on TPs (h2, d4, and d7). The examination of the samples from BSDs showed a less distinctive separation pattern based on the IR dose; however, a sample separation based on TPs with a non-linear transition along the two dimensions of the plot could be discerned, which invoked a level of TP-based similarity in the response in all three sublethal doses. The non-linear correlation of the TPs of BSD samples is suggestive of a multiphase transcriptomic response to sublethal doses, which would explain the stochasticity of responses in this IR range. The differences in responses between BLDs and BSDs that were recognized in the Sammon plot were confirmed by larger numbers of the SDTGs in BLDs (2–5 folds at h2, d4, and d7) relative to those in the BSDs. More importantly, most of the SDTGs were downregulated in BLDs, contrary to the prevalent upregulation of SDTGs in BSDs. Two additional salient features of the transcriptional modulation dynamics differentiate responses to lethal and sublethal IR doses. The first is the stationary regulation of SDTGs in lethal doses where genes common to all TPs represented 59% of the total SDTGs with a steady downregulation of the vast majority, while the percentage of common genes in the same TPs did not exceed 11% in all BSDs. The large difference between the ratios of common SDTGs in BLDs and BSDs was also associated with percentages of TP-specific SDTGs that were larger in BSDs relative to BLDs. The second is that the ratio of up- and downregulated SDTGs in each TP and IR dose did not vary as mice response progressed. These transcription dynamics were more pronounced among the BSDs and were illustrated best by the different transcription peak times in each of the sublethal IR doses (i.e., 1, 3, 6 Gy). Following the changes in the ratios of upregulated and downregulated SDTGs relative to the total SDTGs throughout all TPs in each IR dose uncovered the staggering regulation differences in the BLD and all BSDs, which simplifies distinguishing exposure to lethal from sublethal IR doses as early as h2; it also reveals a delay in the peak of the ratio with increasing dose in sublethal doses, which provides insight into the magnitude of IR dose in the sublethal range. 

The ratios of up/downregulated SDTGs peaked at days 4 and 7 in 1 and 3 Gy then dropped more sharply in 1 Gy relative to 3 Gy, while in 6 Gy, it kept increasing slowly to peak at d21 then dropped sharply at d28. The ratios of the numbers of up/downregulated SDTGs offer a dosimetric tool to assess radiation exposure intensity within the sublethal IR dose range and predict survival. Large percentages of steadily downregulated SDTGs in the early TPs (h2 to d7) were associated with lethal exposure, while a dynamic regulation with large percentages of upregulated SDTGs in the early TPs coincided with lower IR doses and mice survival. The earlier reduction in upregulated SDTGs percentages was associated with lower IR. 

The stochastic nature of the response to lower IR doses is a well-documented hurdle that precludes the prediction of radiation exposure intensity in the sublethal range. In this work, exposure to 3 Gy resulted in numbers of SDTGs that were lower relative to the 1 Gy dose, demonstrating the inability to use SDTGs numbers as a dosimetric tool in IR exposure. A possible explanation of the non-linearity of the numbers of SDTGs in 1, 3, and 6 Gy, particularly the decrease in the number of SDTGs in all TPs of 3 Gy relative to 1 and 6 Gy, is the involvement of multiple types of responses, including activation and/or inactivation, triggered by different IR dose intensities and direct undetected damages to enzymatic or protein synthesis systems that are involved in transcription. Because the number of SDTGs common to all TPs increases and TP-unique SDTGs decrease with increasing IR dose, the ratio of common to unique SDTGs provides an alternative that circumvents the stochasticity and makes the assessment of the IR exposure possible even in the sublethal range. Common to unique SDTGs ratios after the 1, 3, 6, and 20 Gy exposures were 3.2, 2.8, 1.3, and 0.1, respectively, indicating an acceptable dose–response with potential application for estimating the radiation exposure intensity in the stochastic range of IR. Combining the common/unique ratio with the ratio of up/downregulated SDTGs enhances the confidence in the absorbed IR dose estimate and presents a viable tool for survival prediction. 

Tracking the genes that showed transcriptional modulations across TPs and IR doses revealed regulation patterns with potential applications in differentiating lethal and sublethal exposure and supporting a dosimetric tool. Exposure to 20 Gy was associated with transcriptional modulations in a large number of lethal dose-specific genes (390 genes) that were modulated as early as h2 after exposure and retained the same regulation mode until euthanasia. The number of genes specific to sublethal dose exposure was much less mainly due to the highly dynamic and stochastic response resulting in a shorter list of commonly modulated genes among all TPs of the sublethal doses (10 genes). Only three of these ten SDTGs were unique to sublethal exposures; these were calcium- and other metal-binding protein products of the hornerin (Hrnr) gene, which plays an important role in the barrier integrity state and the keratinization of skin and hemopoietic cell differentiation; Cyp2b13/Cyp2b9, which encodes an enzyme with a heme- and ion-binding capacity, oxidoreductase activity, and xenobiotic metabolism that diminishes the damaging effects of oxidative stress; and cystatin A (CSTA), which encodes a stefin that functions as a cysteine protease inhibitor and a precursor for keratinocytes’ cornified cell envelope, essential for the development and homeostasis of the epidermis. Another panel of five genes found commonly, but oppositely, differentially regulated in both lethal and sublethal IR doses at all TPs would serve as an excellent tool in identifying exposure to radiation with insights on survival probability. All five genes at all TPs were upregulated in sublethal (1, 3, and 6 Gy) and downregulated in lethal (20 Gy) doses. The upregulation of these genes favors survival and repair after cell injury. Three of these five genes, namely arylacetamide deacetylase (AADAC), ELOVL fatty acid elongase 4 (ELOVL4), and transmembrane serine protease 4 (TMPRSS4), encode enzymes. The first is involved with heparan sulfate biosynthesis, lipid and xenobiotic metabolic processes, and the positive regulation of triglycerides’ catabolic processes. The second enzyme plays an important role in fatty acid synthesis, cell degeneration, and apoptosis. Disruptions of ELOVL4 synthesis were implicated in dermatological disorders and different types of ataxias. The third enzyme is a protease with peptidase and hydrolase activities involved in the regulation of gene expression and wound healing. The other two genes are SEC14-like lipid binding 4 (SEC14L4), which is a transporter that has lipid and protein binding functions, and the secreted LY6/PLAUR domain-containing 1 (SLURP1) gene, which is thought to encode a secreted protein because it lacks a GPI-anchoring signal sequence. Disruptions in SLURP1 were associated with skin disorders, such as Mal de Meleda disease, and neurological symptoms after IR exposures. The addition of the three sublethal-unique genes (Hrnr, Cyp2b13/Cyp2b9, and CSTA) in combination with a few other genes from the large list of lethal-dose-specific genes commonly modulated in all TPs (390 genes), such as the sharply upregulated transmembrane protein 37 (TMEM37), the sharply downregulated collagen type III alpha 1 chain (COL3A1), and collagen type 1 alpha 2 chain (Table S2), create a robust diagnostic foundation of a device capable of assessing radiation exposure and predicting survivability. Keratin-associated protein 4–7 (Krtap 4–7) was the only gene uniquely differentially transcribed at h2 by all three sublethal doses (FC = 2.265, 2.507, and 2.855 at 1, 3, 6 Gy, respectively). The protein encoded by Krtap 4–7 is a member of the ultrahigh sulfur subfamily of the keratin-associated protein (KAP) family which forms a matrix of keratin intermediate filaments in hair fibers. The short-lived transcriptional modulation of Krtap 4–7 might have applications in determining previous IR exposure in radiation forensics. 

Lists of SDTGs included large numbers of genes encoding keratins, collagens, cytokines, enzymes, growth factors, transcription factors, transporters, and ion channels among other functions. The opposite regulation mode between lethal and sublethal doses with predominant gene downregulation in lethal doses and the steady regulation of SDTGs in all TPs of the lethal dose relative to changing regulation in the TPs of sublethal doses were the main characteristics of gene transcription modulations. These differences between lethal and sublethal doses predicted the inactivation of lipid synthesis, fatty acid metabolism, cellular movement, leukocyte migration, cell activation, and cell survival in lethal doses. These functions were generally predicted to be activated in sublethal IR doses during h2, d4, and/or d7 based on the dose level in what seems to be part of an early repair response. Most of these functions returned to a normal level or exhibited an inactivated state simulating responses after lethal dose exposure. Similarly, organismal death, disorder and loss of hair, and morbidity or mortality functions were all predicted to be activated in lethal and inactivated in sublethal IR doses. Interestingly, gene regulation and the associated activation status of these functions in sublethal doses for some genes shifted to simulate that in sublethal doses at d21 and d28, especially in the 6 Gy dose, suggesting a second stressful phase of response after exposure to sublethal IR dose effects. The impact of the late-phase similarities in transcriptional and biofunctional modulations between sublethal/lethal doses on animal survival was beyond the scope of this work. 

Pathway enrichments aiming to identify the affected pathways in the lethal dose showed 22 significantly modulated pathways, with the majority being inactivated (21/22). More than half (12/22) were steadily inactivated at all three TPs, and three pathways were inactivated in both d4 and d7. The other seven pathways were inactivated at d4 (six pathways), and only the PI3K/AKT pathway was activated at h2. The activation of this pathway was mainly due to the upregulation of BCL2-like 1 (BCL2-L1) and the eukaryotic translation initiation factor 4E binding protein 1 (EIF4EBP1) genes. The product of the BCL2-L1 gene is a BCL2 family member that forms hetero- or homodimers and acts as an anti- or pro-apoptotic regulator, while the other gene encodes a repressor protein of the eukaryotic translation initiation factor 4E (eIF4E) to repress translation. The regulation of these two genes among others in the pathway is consistent with the lethargic biofunctions in the BLD.

The overwhelming extended inactivation of the pathways reflects the dominant downregulation in SDTGs and highlights the early commitment to death after exposure to a 20 Gy IR dose. A comparison of the state of the top affected pathways after a lethal dose with that in sublethal doses confirmed the staggered response in survivable and non-survivable IR doses. All pathways identified from the analysis of the first three TPs in lethal and sublethal doses had the opposite activation status, with the majority being activated after sublethal doses and inactivated after a lethal dose. The identified pathways provided important insights into the mechanisms underlying well-documented reactions of the skin, immune, hematological, neurological, endothelial, and to a lesser extent the lung and circulatory systems to radiation exposure. Results from the pathways analysis show that the identified significantly affected pathway plays a role in the regulation of responses in the immune system, hematological development, tissue detoxification, skin reactions and fibrosis in the skin and other organs, lipids and cholesterol turnover, cytoskeletal organization, and related cell mobility, differentiation, survival, and barrier functions. The focus of this report was to introduce indicative transcriptomic patterns and describe their potential applications in radiation diagnosis and prognosis. Future work will target additional investigations of the pathways and SDTGs introduced in this work for their independent role in radiation-induced damage using different types of tissues and organs. Additional work is needed to validate the findings of this work in a larger population of mice with a wide age range, as the response to radiation tends to vary significantly based on age and comorbidities.

## Figures and Tables

**Table 1:** Distribution of total SDTGs from global analysis of data in the study (all TPs and IR doses).

IR Dose	Union in All TPs	Common to All TPs	Number of TP-Unique Molecules				
			2 h	D4	D7	D21	D28
1 Gy	1665	0	37	16	19	8	5
			2.22%	0.96%	1.14%	0.48%	0.3%
3 Gy			4	6	2	28	32
			0.24%	0.36%	0.12%	1.68%	1.92%
6 Gy			4	43	55	35	18
			0.24%	2.58%	3.3%	2.1%	1.08%
20 Gy			34	50	58	NA	NA
			2.04%	3%	3.48%		

**Table 2:** Distribution of SDTGs on time points using independent analysis for each IR dose.

IR Dose	Union in All TPs	Common to All TPs	Number of TP-Unique Molecules				
			2 h	d4	d7	d21	d28
1 Gy	619	1	125	101	26	51	62
		0.16%	20.2%	16.3%	4.2%	8.2%	10%
3 Gy	411	0	61	35	20	99	82
			14.8%	8.5%	4.9%	24.1%	20%
6 Gy	765	4	47	84	173	52	88
		0.52%	6.1%	11.0%	22.6%	6.8%	11.5%
20 Gy	1038	609	57	80	89	NA	NA
		58.6%	5.5%	7.7%	8.6%		

**Table 3:** List of time-point-unique SDTGs (*p* < 0.05 and FC larger than 3.5 FC) from independent analysis of modulated genes at each IR dose. Time points that did not contain genes passing the FC threshold are not included in the table.

IR Dose	Time Point	Symbol	Upregulated		Downregulated		
			Gene Name	Expr FC	Symbol	Gene Name	Expr FC
1 Gy	2 h	TRIM63	tripartite motif containing 63	4.607	KRT31	keratin 31	−3.736
		MYLK4	myosin light chain kinase family member 4	4.488			
		MSTN	Myostatin	3.841			
	d4	Stfa2/Stfa2l1	stefin A2	5.821	no genes < −3.5		
		S100A9	S100 calcium-binding protein A9	3.711			
	d7	D030036P13Rik	RIKEN cDNA D030036P13 gene	5.008	no genes < −3.5		
		4930556J24Rik	RIKEN cDNA 4930556J24 gene	4.293			
		MKNK1	MAPK interacting serine/threonine kinase 1	4.078			
		NET1	neuroepithelial cell transforming 1	4.051			
		LIN7A	lin-7 homolog A, crumbs cell polarity complex component	3.728			
	d21	PRR9	proline rich 9	4.542			
	d28	Kap	kidney androgen-regulated protein	4.359	PPP1R3C	protein phosphatase 1 regulatory subunit 3C	−3.856
					KRT71	keratin 71	−6.13
3 Gy	2 h	Defb8	defensin beta 8	4.342	no genes		
	d7	PON1	paraoxonase 1	3.654	no genes		
	d21	NR4A1	nuclear receptor subfamily 4 group A member 1	4.566	MLANA	melan-A	−3.711
		FOSB	FosB proto-oncogene, AP-1 transcription factor subunit	3.909			
		FOS	Fos proto-oncogene, AP-1 transcription factor subunit	3.734	LGR5	leucine-rich repeat containing G protein-coupled receptor 5	−3.862
		CCL4	C-C motif chemokine ligand 4	3.557			
	d28	GJB2	gap junction protein beta 2	5.18	PPP1R3C	protein phosphatase 1 regulatory subunit 3C	−3.942
6 Gy	d4	PLAC8	placenta-associated 8	5.227	no genes < −3.5		
		CCL2	C-C motif chemokine ligand 2	4.079			
		FCGR1A	Fc fragment of IgG receptor Ia	3.649			
	d7	Stfa2/Stfa2l1	stefin A2	11.053	Krtap16-3	keratin-associated protein 16-3	−3.576
					1110025L11Rik	RIKEN cDNA 1110025L11 gene	−3.597
		KLHL36	kelch-like family member 36	5.555	KRT34	keratin 34	−3.734
					FBP1	fructose-bisphosphatase 1	−3.755
		CKMT2	creatine kinase, mitochondrial 2	4.435	Krtap8-1	keratin-associated protein 8-1	−3.864
					Krtap22-2	keratin-associated protein 22-2	−3.938
		KLK6	kallikrein-related peptidase 6	4.3	Gm10229	predicted gene 10229	−4.033
					PRR9	proline rich 9	−4.416
		KRT6B	keratin 6B	3.847	Krtap19-1	keratin-associated protein 19-1	−5.359
					KRT27	keratin 27	−5.532
					KRT25	keratin 25	−8.664
	d21	CHRNA1	cholinergic receptor nicotinic alpha 1 subunit	5.57	no genes < −3.5		
		FOS	Fos proto-oncogene, AP-1 transcription factor subunit	5.228			
		CHRNG	cholinergic receptor nicotinic gamma subunit	4.627			
		HSPB7	heat shock protein family B (small) member 7	4.321			
		ANKRD1	ankyrin repeat domain 1	4.161			
		MYH3	myosin heavy chain 3	3.54			
	d28	no genes > 3.5			Fam25c	family with sequence similarity 25, member C	−3.683
					Krt10	keratin 10	−3.758
					PPP1R3C	protein phosphatase 1 regulatory subunit 3C	−3.964
20 Gy	2 h	no genes > 3.5			KRT28	keratin 28	−3.725
	d4				ENO3	enolase 3	−3.848
					PPP1R3C	protein phosphatase 1 regulatory subunit 3C	−4.105

**Table 4:** Time point-based comparison of SDTGs’ (*p* < 0.05 and FC ≥ 2) distribution among responses to all IR doses.

Time Point	Union	# of Common SDTGs	# of IR Dose-Unique SDTGs			
			1 Gy	3 Gy	6 Gy	20 Gy
2 h	1096	17 1.55%	83	14	36	702
			7.57%	1.27%	3.28%	64.05%
d4	1079	24 2.22%	78	15	107	620
			7.22%	1.39%	9.91%	57.46%
d7	1121	16 1.42%	34	7	179	666
			3.03%	0.62%	15.96%	59.41%
d21	314	5	47	76	118	NA
		−1.47%	14.96%	24.20%	37.57%	
d28	322	19	21	66	112	NA
		5.90%	6.52%	20.49%	34.78%	

**Table 5:** List of SDTGs (*p* < 0.05, FC ≥ 2 and average > 3 FC) common to all IR doses at each time point.

Time Point	Symbol	Molecule Name	Expr Fold Change				Location	Type(s)
			(1 Gy)	(3 Gy)	(6 Gy)	(20 Gy)		
2 h	AADAC *	arylacetamide deacetylase	2.41	2.86	2.89	−7.09	Cytoplasm	enzyme
	ABRA	actin-binding Rho activating protein	5.57	2.69	2.93	4.37	Cytoplasm	other
	ACOXL	acyl-CoA oxidase-like	2.57	2.75	3.08	−3.92	Other	enzyme
	ELOVL4 *	ELOVL fatty acid elongase 4	2.09	2.10	2.32	−7.36	Cytoplasm	enzyme
	ELOVL6	ELOVL fatty acid elongase 6	2.03	2.20	2.34	−6.34	Cytoplasm	enzyme
	FA2H	fatty acid 2-hydroxylase	2.34	2.32	2.38	−20.85	Cytoplasm	enzyme
	MYH2	myosin heavy chain 2	4.55	2.74	4.69	−3.49	Cytoplasm	enzyme
	SEC14L4 *	SEC14-like lipid binding 4	2.58	2.38	2.72	−6.28	Other	transporter
	Wfdc3	WAP four-disulfide core domain 3	2.30	2.23	2.78	−9.16	Other	other
d4	ACOXL	acyl-CoA oxidase-like	3.11	2.12	3.11	−4.04	Other	enzyme
	TMPRSS4	transmembrane serine protease 4	4.94	3.07	2.05	−2.69	Cytoplasm	peptidase
	Serpina3b/Serpina3j	serine (or cysteine) peptidase inhibitor, clade A (alpha-1 antiproteinase, antitrypsin), member 3J	3.53	2.22	2.41	−4.66	Extracellular Space	other
	HBB	hemoglobin subunit beta	2.31	−2.12	−2.83	−6.06	Cytoplasm	transporter
	ELOVL6	ELOVL fatty acid elongase 6	2.83	2.04	3.22	−5.49	Cytoplasm	enzyme
	ELOVL3	ELOVL fatty acid elongase 3	2.72	2.06	2.90	−6.43	Cytoplasm	enzyme
	SEC14L4 *	SEC14-like lipid binding 4	3.46	2.18	2.88	−6.46	Other	transporter
	ELOVL4 *	ELOVL fatty acid elongase 4	3.48	2.57	3.05	−5.93	Cytoplasm	enzyme
	AADAC *	arylacetamide deacetylase	3.89	2.49	2.43	−7.44	Cytoplasm	enzyme
	Wfdc3	WAP four-disulfide core domain 3	2.91	2.25	2.73	−8.40	Other	other
	RNASE2	ribonuclease A family member 2	2.93	4.32	4.32	−10.35	Cytoplasm	enzyme
	S100A9	S100 calcium-binding protein A9	3.71	5.80	3.00	11.25	Cytoplasm	other
	FA2H	fatty acid 2-hydroxylase	3.78	2.29	3.45	−16.57	Cytoplasm	enzyme
d7	RNASE2	ribonuclease A family member 2	3.23	3.20	6.71	−12.54	Cytoplasm	enzyme
	AADAC *	arylacetamide deacetylase	2.86	2.31	6.31	−8.54	Cytoplasm	enzyme
	SEC14L4 *	SEC14-like lipid binding 4	3.11	2.17	6.51	−6.50	Other	transporter
	Aldh3b2	aldehyde dehydrogenase 3 family, member B2	2.06	2.19	4.40	−9.45	Cytoplasm	enzyme
	ELOVL4 *	ELOVL fatty acid elongase 4	2.73	2.32	5.50	−6.79	Cytoplasm	enzyme
	AQP3	aquaporin 3 (Gill blood group)	2.84	2.82	6.87	−2.84	Plasma Membrane	transporter
	Defb6	defensin beta 6	2.47	2.00	5.36	−5.33	Extracellular Space	other
	Sdr16c6	short chain dehydrogenase/reductase family 16C, member 6	2.59	2.58	4.89	−3.77	Other	other
	SLURP1	secreted LY6/PLAUR domain containing 1	3.01	2.31	4.33	−3.29	Extracellular Space	cytokine
	Wfdc21	WAP four-disulfide core domain 21	2.67	2.24	4.43	−2.70	Extracellular Space	other
d21	FLNC	filamin C	2.81	2.95	5.74	NA	Cytoplasm	other
d28	PPP1R3C	protein phosphatase 1 regulatory subunit 3C	−3.86	−3.94	−3.96	NA	Cytoplasm	phosphatase
	Mup1 (includes others)	major urinary protein 1	−3.11	−3.44	−3.81	NA	Extracellular Space	other
	Kap	kidney androgen-regulated protein	4.36	2.50	2.96	NA	Extracellular Space	other

Genes denoted with * are common to all 3 TPs in 1, 3, 6, 20Gy with opposite regulation (sharply downregulated in lethal and upregulated in sublethal doses).

**Table 6:** Radiation-induced transcriptional modulations using SDTG (*p* < 0.05 and FC ≥ 2) distribution in the first three time points and comparison of modulations in lethal and sublethal IR doses.

Union (ALL 3 TPs)	Common SDTGs (ALL 3 TPs)	Time Point	Union	TP-Common SDTGs	IR Dose-Unique SDTGs			
					1Gy	3Gy	6Gy	20Gy
1499	5	2h	1096	17 1.55%	83	14	36	702
					7.57%	1.27%	3.28%	64.05%
		d4	1079	24 2.22%	78	15	107	620
					7.22%	1.39%	9.91%	57.46%
		d7	1121	16 1.42%	34	7	179	666
					3.03%	0.62%	15.96%	59.41%

**Table 7:** Commonly modulated genes in all time points after exposure to lethal and sublethal IR doses. Note the opposite response in gene regulation to sublethal and lethal doses.

Symbol	Entrez Gene Name	1 Gy-2 h	1 Gy-d4	1 Gy-d7	3 Gy-2 h	3 Gy-d4	3 Gy-d7	6 Gy-2 h	6 Gy-d4	6 Gy-d7	20 Gy-2 h	20 Gy-d4	20 Gy-d7	Location	Function
AADAC	arylacetamide deacetylase	2.41	3.89	2.86	2.86	2.49	2.31	2.89	2.43	6.31	−7.09	−7.44	−8.54	Cytoplasm	enzyme
ELOVL4	ELOVL fatty acid elongase 4	2.09	3.48	2.73	2.10	2.57	2.32	2.32	3.05	5.50	−7.36	−5.93	−6.79	Cytoplasm	enzyme
SEC14L4	SEC14-like lipid binding 4	2.58	3.46	3.11	2.38	2.18	2.17	2.72	2.88	6.51	−6.28	−6.46	−6.50	Other	transporter
SLURP1	secreted LY6/PLAUR domain containing 1	2.29	3.33	3.01	2.07	3.10	2.31	2.21	2.95	4.33	−3.08	−2.52	−3.29	Extracellular Space	cytokine
TMPRSS4	transmembrane serine protease 4	3.01	4.94	2.39	3.30	3.07	3.11	2.08	2.05	3.35	−2.67	−2.69	−2.77	Cytoplasm	peptidase

## Data Availability

Minimum information about a microarray experiment (MIAME)-compliant intensity, quality, and normalized ratio data for this series of experiments have been deposited in the gene expression omnibus (GEO) database maintained by the National Center for Biotechnology Information (accession No. GSE185149).

## Supplementary Materials

The following supporting information can be downloaded at: https://www.mdpi.com/article/10.3390/cimb44080254/s1, Table S1: Time point-based comparison of SDTGs (*p* < 0.05 and FC ≥ 2) distribution among sublethal IR doses, Table S2: Top SDTGs in BLDs with an average fold change regulation larger than ten (Ave FC > 10 and *p* < 0.05) in all time points. Figure S1: Significantly (Abs z-score ≥ 2 and -log *p* ≥ 1.3) modulated pathways in at least one time point from the analysis of SDTGs ( *p* ≤ 0.05 and Abs FC ≥ 2) at h2, d4, and d7 TPs in lethal (20Gy) and sublethal IR doses (1, 3, 6Gy). Color of squares ranges according to modulation intensity from dark blue for inactivation to dark red for activation prediction based on z-score values

## References

[1] Thomas G.A., Symonds P. (2016). Radiation Exposure and Health Effects—Is it Time to Reassess the Real Consequences?. Clin. Oncol..

[2] Merrifield M., Kovalchuk O. (2013). Epigenetics in radiation biology: A new research frontier. Front. Genet..

[3] Lee K.-F., Chen Y.-C., Hsu P.W.-C., Liu I.Y., Wu L.S.-H. (2014). MicroRNA expression profiling altered by variant dosage of radiation exposure. BioMed Res. Int..

[4] Coy S.L., Cheema A.K., Tyburski J.B., Laiakis E.C., Collins S.P., Fornace A.J. (2011). Radiation metabolomics and its potential in biodosimetry. Int. J. Radiat. Biol..

[5] Donnelly E.H., Nemhauser J.B., Smith J.M., Kazzi Z.N., Farfán E.B., Chang A.S., Naeem S.F. (2010). Acute Radiation Syndrome: Assessment and Management. South Med. J..

[6] Nepper-Christensen S., Heslet L., Bay C. (2012). Acute radiation syndrome (ARS)—Treatment of the reduced host defense. Int. J. Gen. Med..

[7] Grosovsky A.J., Parks K.K., Giver C.R., Nelson S.L. (1996). Clonal analysis of delayed karyotypic abnormalities and gene mutations in radia-tion-induced genetic instability. Mol. Cell. Biol..

[8] Roy L., Gruel G., Vaurijoux A. (2009). Cell response to ionising radiation analysed by gene expression patterns. Ann. Ist. Super. Sanita.

[9] Riecke A., Rufa C.G., Cordes M., Hartmann J., Meineke V., Abend M. (2012). Gene Expression Comparisons Performed for Biodosimetry Purposes on In Vitro Peripheral Blood Cellular Subsets and Irradiated Individuals. Radiat. Res..

[10] Rivina L., Davoren M.J., Schiestl R.H. (2016). Mouse models for radiation-induced cancers. Mutagenesis.

[11] Rivina L., Schiestl R. (2012). Mouse Models for Efficacy Testing of Agents against Radiation Carcinogenesis—A Literature Review. Int. J. Environ. Res. Public Health.

[12] Seed T.M., Inal C.E., Singh V.K. (2014). Radioprotection of hematopoietic progenitors by low dose amifostine prophylaxis. Int. J. Radiat. Biol..

[13] Singh V.K., Newman V.L., Romaine P.L., Hauer-Jensen M., Pollard H.B. (2015). Use of biomarkers for assessing radiation injury and efficacy of countermeasures. Expert Rev. Mol. Diagn..

[14] Swartz H.M., Flood A.B., Gougelet R.M., Rea M.E., Nicolalde R.J., Williams B.B. (2010). A critical assessment of biodosimetry methods for large-scale incidents. Health Phys..

[15] Wang X., Zhang J., Fu J., Wang J., Ye S., Liu W., Shao C. (2015). Role of ROS-mediated autophagy in radiation-induced bystander effect of hepatoma cells. Int. J. Radiat. Biol..

[16] Singh V.K., Fatanmi O.O., Singh P.K., Whitnall M.H. (2012). Role of radiation-induced granulocyte colony-stimulating factor in recovery from whole body gamma-irradiation. Cytokine.

[17] Singh V.K., Ducey E.J., Fatanmi O.O., Singh P.K., Brown D.S., Purmal A., Shakhova V.V., Gudkov A.V., Feinstein E., Shakhov A. (2011). CBLB613: A TLR 2/6 Agonist, Natural Lipopeptide of *Mycoplasma arginini*, as a Novel Radiation Countermeasure. Radiat. Res..

[18] Singh V.K., Christensen J., Fatanmi O.O., Gille D., Ducey E.J., Wise S.Y., Karsunky H., Sedello A.K. (2012). Myeloid Progenitors: A Radiation Countermeasure that is Effective when Initiated Days after Irradiation. Radiat. Res..

[19] Singh V.K., Brown D.S., Kao T.C. (2010). Alpha-tocopherol succinate protects mice from gamma-radiation by induction of granulo-cyte-colony stimulating factor. Int. J. Radiat. Biol..

[20] Krivokrysenko V.I., Shakhov A.N., Singh V., Bone F., Kononov Y., Shyshynova I., Cheney A., Maitra R.K., Purmal A., Whitnall M.H. (2012). Identification of Granulocyte Colony-Stimulating Factor and Interleukin-6 as Candidate Biomarkers of CBLB502 Efficacy as a Medical Radiation Countermeasure. J. Pharmacol. Exp. Ther..

[21] Ha C.T., Li X.-H., Fu D., Moroni M., Fisher C., Arnott R., Srinivasan V., Xiao M. (2014). Circulating Interleukin-18 as a Biomarker of Total-Body Radiation Exposure in Mice, Minipigs, and Nonhuman Primates (NHP). PLoS ONE.

[22] Thomas G.K., Trankle C.R., Carbone S., Billingsley H., Van Tassell B.W., Evans R.K., Garten R., Weiss E., Abbate A. (2020). Increased C-reactive protein is associated with the severity of thoracic radiothera-py-induced cardiomyopathy. Cardio-Oncology.

[23] Bertho J.-M., Demarquay C., Frick J., Joubert C., Arenales S., Jacquet N., Sorokine-Durm I., Chau Q., Lopez M., Aigueperse J. (2001). Level of Flt3-ligand in plasma: A possible new bio-indicator for radiation-induced aplasia. Int. J. Radiat. Biol..

[24] Sproull M., Camphausen K. (2016). State-of-the-Art Advances in Radiation Biodosimetry for Mass Casualty Events Involving Radiation Exposure. Radiat. Res..

[25] Hu S., Blakely W.F., Cucinotta F.A. (2015). HEMODOSE: A Biodosimetry Tool Based on Multi-type Blood Cell Counts. Health Phys..

[26] Wong K.F., Siu L.L.P., Ainsbury E., Moquet J. (2013). Cytogenetic biodosimetry: What it is and how we do it. Hong Kong Med. J..

[27] Haber J.E., Thorburn P.C., Rogers D. (1984). Meiotic and mitotic behavior of dicentric chromosomes in saccharomyces cerevisiae. Genetics.

[28] Kravtsov V., Fedortseva R., Starkova Y., Yartseva N., Nikiforov A. (2000). Tailed nuclei and dicentric chromosomes in irradiated subjects. Appl. Radiat. Isot..

[29] Singh V.K., Santiago P.T., Simas M., Garcia M., Fatanmi O.O., Wise S.Y., Seed T.M. (2018). Acute radiation syndrome: An update on biomarkers for radiation injury. J. Radiat. Cancer Res..

[30] Mah L.J., El-Osta A., Karagiannis T.C. (2010). gammaH2AX: A sensitive molecular marker of DNA damage and repair. Leukemia.

[31] Redon C.E., Nakamura A.J., Martin O.A., Parekh P.R., Weyemi U.S., Bonner W.M. (2011). Recent developments in the use of gamma-H2AX as a quantitative DNA dou-ble-strand break biomarker. Aging.

[32] Ivashkevich A., Redon C.E., Nakamura A.J., Martin R.F., Martin O.A. (2012). Use of the gamma-H2AX assay to monitor DNA damage and repair in trans-lational cancer research. Cancer Lett..

[33] Markova E., Torudd J., Belyaev I. (2011). Long time persistence of residual 53BP1/gamma-H2AX foci in human lymphocytes in rela-tionship to apoptosis, chromatin condensation and biological dosimetry. Int. J. Radiat. Biol..

[34] Bourton E.C., Plowman P.N., Smith D., Arlett C.F., Parris C.N. (2011). Prolonged expression of the gamma-H2AX DNA repair biomarker correlates with excess acute and chronic toxicity from radiotherapy treatment. Int. J. Cancer.

[35] Jin Y.W., Na Y.J., Lee Y.J., An S., Lee J.E., Jung M., Kim H., Nam S.Y., Kim C.S., Yang K.H. (2008). Comprehensive analysis of time- and dose-dependent patterns of gene expression in a human mes-enchymal stem cell line exposed to low-dose ionizing radiation. Oncol. Rep..

[36] Aypar U., Morgan W.F., Baulch J.E. (2011). Radiation-induced epigenetic alterations after low and high LET irradiations. Mutat. Res. Mol. Mech. Mutagen..

[37] Aypar U., Morgan W.F., Baulch J.E. (2011). Radiation-induced genomic instability: Are epigenetic mechanisms the missing link?. Int. J. Radiat. Biol..

[38] Kaup S., Grandjean V., Mukherjee R., Kapoor A., Keyes E., Seymour C.B., Mothersill C.E., Schofield P.N. (2006). Radiation-induced genomic instability is associated with DNA methylation changes in cultured human keratinocytes. Mutat. Res. Mol. Mech. Mutagen..

[39] Pernot E., Hall J., Baatout S., Benotmane M.A., Blanchardon E., Bouffler S., El Saghire H., Gomolka M., Guertler A., Harms-Ringdahl M. (2012). Ionizing radiation biomarkers for potential use in epidemiological studies. Mutat. Res. Rev. Mutat. Res..

[40] Pannkuk E.L., Fornace A.J., Laiakis E.C. (2017). Metabolomic applications in radiation biodosimetry: Exploring radiation effects through small molecules. Int. J. Radiat. Biol..

[41] Pannkuk E.L., Laiakis E.C., Authier S., Wong K., Fornace A.J. (2015). Global Metabolomic Identification of Long-Term Dose-Dependent Urinary Biomarkers in Nonhuman Primates Exposed to Ionizing Radiation. Radiat. Res..

[42] Laiakis E.C. (2019). Metabolomic Applications in Radiation Biodosimetry. Methods Mol. Biol..

[43] Hyduke D.R., Laiakis E.C., Li H.H., Fornace A.J. (2013). Identifying radiation exposure biomarkers from mouse blood transcriptome. Int. J. Bioinform. Res. Appl..

[44] Almond P.R., Biggs P.J., Coursey B.M., Hanson W.F., Huq M.S., Nath R., Rogers D.W. (1999). AAPM’s TG-51 protocol for clinical reference dosimetry of high-energy photon and electron beams. Med. Phys..

[45] Ritchie M.E., Belinda P., Wu D., Hu Y., Law C.W., Shi W., Smyth G.K. (2015). Limma powers differential expression analyses for RNA-sequencing and microarray studies. Nucleic Acids Res..

