# Supplementary material for: Transcriptomes of Wet Skin Biopsies Predict Outcomes after Ionizing Radiation Exposure with Potential Dosimetric Applications in a Mouse Model
Source: Curr Issues Mol Biol. 2022 Aug 18;44(8):3711–34. doi: 10.3390/cimb44080254 (PMC9406351; doi:10.3390/cimb44080254)
Supplement: Supplementary file 1 [file cimb-44-00254-s001.zip › cimb-1836236-supplementary.pdf]

Supplementary Table S1. Time point-based Comparison of SDTGs ($p < 0.05$ and FC ≥ 2) distribution among sublethal IR doses

Time point	Union	Common in all Doses	Dose-unique (comparison of BSDs only)		
			1Gy	3Gy	6Gy
2h	394	58<br>*14.72%*	117<br>*29.69%*	23<br>*5.83%*	58<br>*14.72%*
d4	459	48<br>*10.45%*	159<br>*34.64%*	19<br>*4.13%*	137<br>*29.84%*
d7	455	26<br>5.71%	42<br>9.23%	9<br>1.97%	287<br>63.07%
d21	314	5<br>*1.47%*	47<br>*14.96%*	76<br>*24.20%*	118<br>*37.57%*
d28	322	19<br>*5.90%*	21<br>*6.52%*	66<br>*20.49%*	112<br>*34.78%*

Supplementary Table S2. Top SDTGs in BLD with an average fold change regulation larger than ten (Ave FC > 10 and $p < 0.05$) in all time points.

Symbol	Entrez Gene Name	Fold change (FC)			Location	Function
		h2	d4	d7		
Ctla2a/Ctla2b	cytotoxic T lymphocyte-associated protein 2 alpha	9.981	14.219	14.814	Plasma Membrane	other
S100A9	S100 calcium binding protein A9	7.161	11.249	12.778	Cytoplasm	other
TMEM37*	transmembrane protein 37	9.533	10.832	10.335	Plasma Membrane	ion channel
COL3A1*	collagen type III alpha 1 chain	-31.753	-26.584	-47.74	Extracellular Space	other
COL1A1	collagen type I alpha 1 chain	-24.315	-23.331	-35.277	Extracellular Space	other
COL1A2*	collagen type I alpha 2 chain	-22.209	-20.323	-31.159	Extracellular Space	other
FA2H	fatty acid 2-hydroxylase	-20.851	-16.568	-23.311	Cytoplasm	enzyme
Mup1 (includes others)	major urinary protein 1	-17.097	-15.839	-20.242	Extracellular Space	other
DHCR24	24-dehydrocholesterol reductase	-14.088	-12.843	-14.346	Cytoplasm	enzyme
KRT1	keratin 1	-12.075	-11.79	-14.346	Cytoplasm	other
LUM	lumican	-12.706	-9.105	-13.944	Extracellular Space	other
RNASE2	ribonuclease A family member 2	-11.398	-10.353	-12.541	Cytoplasm	enzyme
KRT79	keratin 79	-11.132	-10.281	-10.57	Extracellular Space	other
CIDEA	cell death inducing DFFA like effector a	-11.378	-9.54	-10.169	Cytoplasm	other
MGLL	monoglyceride lipase	-9.456	-9.85	-11.687	Plasma Membrane	enzyme
KRT75*	keratin 75	-10.52	-9.371	-10.211	Cytoplasm	other

*Denote genes that are unique to the 20Gy lethal dose (i.e., not significantly differentially regulated in any sublethal doses).

Supplementary Figure S1. Significantly (Abs z-score ≥ 2 and -log p ≥ 1.3) modulated pathways in at least one time point from the analysis of SDTGs ($p \leq 0.05$ and Abs FC ≥ 2) at h2, d4, and d7 TPs in lethal (20Gy) and sublethal IR doses (1, 3, 6Gy). Color of squares ranges according to modulation intensity from dark blue for inactivation to dark red for activation prediction based on z-score values.

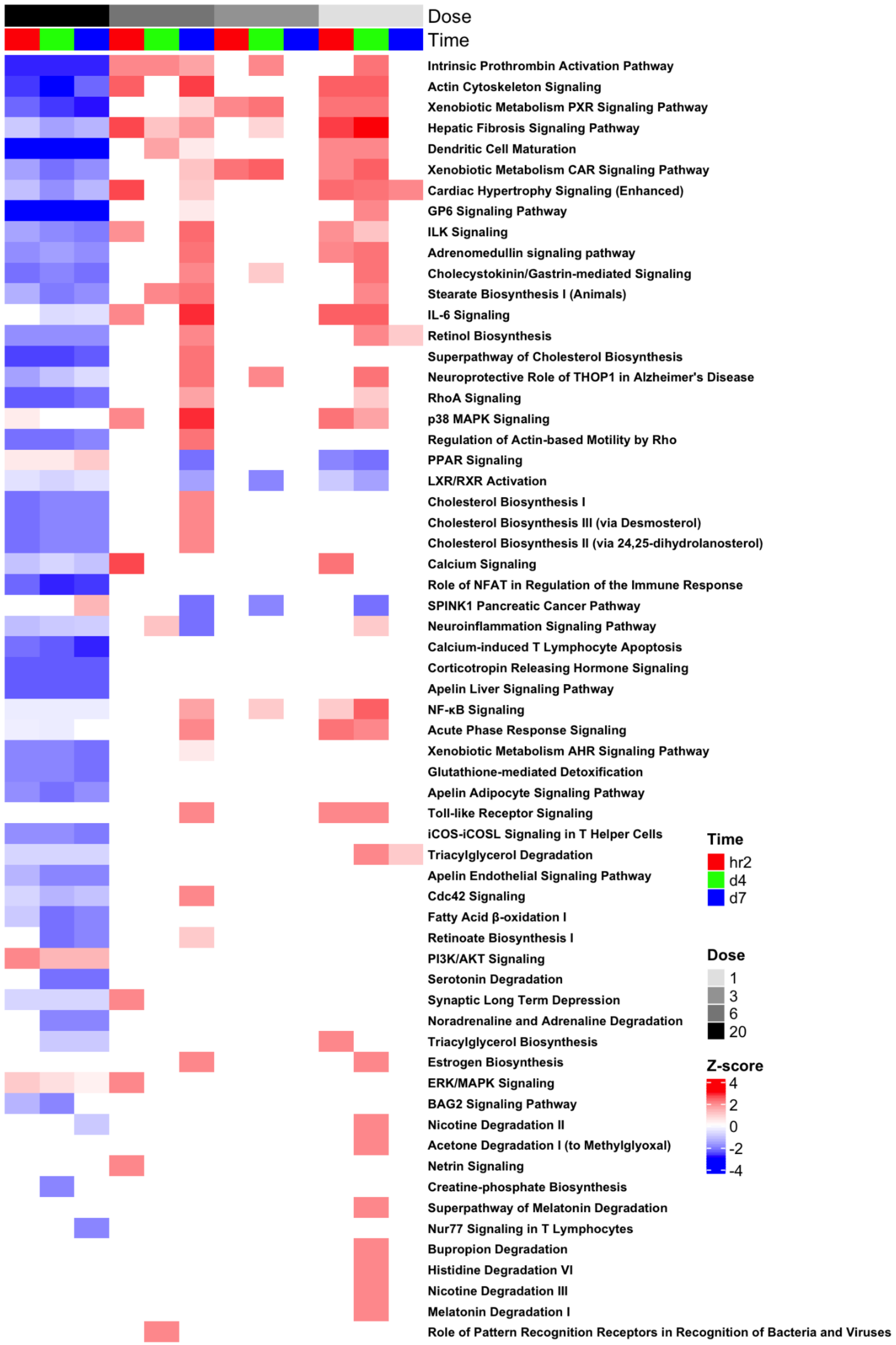
Dose
Time
Intrinsic Prothrombin Activation Pathway
Actin Cytoskeleton Signaling
Xenobiotic Metabolism PXR Signaling Pathway
Hepatic Fibrosis Signaling Pathway
Dendritic Cell Maturation
Xenobiotic Metabolism CAR Signaling Pathway
Cardiac Hypertrophy Signaling (Enhanced)
GP6 Signaling Pathway
ILK Signaling
Adrenomedullin signaling pathway
Cholecystokinin/Gastrin-mediated Signaling
Stearate Biosynthesis I (Animals)
IL-6 Signaling
Retinol Biosynthesis
Superpathway of Cholesterol Biosynthesis
Neuroprotective Role of THOP1 in Alzheimer's Disease
RhoA Signaling
p38 MAPK Signaling
Regulation of Actin-based Motility by Rho
PPAR Signaling
LXR/RXR Activation
Cholesterol Biosynthesis I
Cholesterol Biosynthesis III (via Desmosterol)
Cholesterol Biosynthesis II (via 24,25-dihydrolanosterol)
Calcium Signaling
Role of NFAT in Regulation of the Immune Response
SPINK1 Pancreatic Cancer Pathway
Neuroinflammation Signaling Pathway
Calcium-induced T Lymphocyte Apoptosis
Corticotropin Releasing Hormone Signaling
Apelin Liver Signaling Pathway
NF-κB Signaling
Acute Phase Response Signaling
Xenobiotic Metabolism AHR Signaling Pathway
Glutathione-mediated Detoxification
Apelin Adipocyte Signaling Pathway
Toll-like Receptor Signaling
iCOS-iCOSL Signaling in T Helper Cells
Triacylglycerol Degradation
Apelin Endothelial Signaling Pathway
Cdc42 Signaling
Fatty Acid β-oxidation I
Retinoate Biosynthesis I
PI3K/AKT Signaling
Serotonin Degradation
Synaptic Long Term Depression
Noradrenaline and Adrenaline Degradation
Triacylglycerol Biosynthesis
Estrogen Biosynthesis
ERK/MAPK Signaling
BAG2 Signaling Pathway
Nicotine Degradation II
Acetone Degradation I (to Methylglyoxal)
Netrin Signaling
Creatine-phosphate Biosynthesis
Superpathway of Melatonin Degradation
Nur77 Signaling in T Lymphocytes
Bupropion Degradation
Histidine Degradation VI
Nicotine Degradation III
Melatonin Degradation I
Role of Pattern Recognition Receptors in Recognition of Bacteria and Viruses
Time
hr2
d4
d7
Dose
1
3
6
20
Z-score
4
2
0
-2
-4
